# Glomerular Kidney Diseases in the Single-Cell Era

**DOI:** 10.3389/fmed.2021.761996

**Published:** 2021-10-29

**Authors:** Khun Zaw Latt, Jurgen Heymann, Teruhiko Yoshida, Jeffrey B. Kopp

**Affiliations:** Kidney Disease Section, Kidney Diseases Branch, National Institute of Diabetes and Digestive and Kidney Diseases, National Institutes of Health, Bethesda, MD, United States

**Keywords:** glomerular diseases, single cell genomics, precision medicine, immunity and inflammation, urine biomarkers

## Abstract

Recent advances in single-cell technology have enabled investigation of genomic profiles and molecular crosstalk among individual cells obtained from tissues and biofluids at unprecedented resolution. Glomerular diseases, either primary or secondary to systemic diseases, often manifest elements of inflammation and of innate and adaptive immune responses. Application of single-cell methods have revealed cellular signatures of inflammation, cellular injury, and fibrosis. From these signatures, potential therapeutic targets can be inferred and in theory, this approach might facilitate identification of precision therapeutics for these diseases. Single-cell analyses of urine samples and skin lesions from patients with lupus nephritis and of urine samples from patients with diabetic nephropathy and focal segmental glomerulosclerosis have presented potential novel approaches for the diagnosis and monitoring of disease activity. These single-cell approaches, in contrast to kidney biopsy, are non-invasive and could be repeated multiple times as needed.

## Introduction

Over the past decade, advances in technology have revolutionized our understanding of the genetic and molecular bases of glomerular diseases. For example, transcriptomics studies investigating the genome-wide gene expression profiles of diseased tissues identified candidate genes and pathways upregulated or activated in nephrotic diseases (1, 2). However, due to the lack of spatial and temporal resolution, it is usually not possible to define the exact cellular origins and states of transcriptomics signatures in tissues. Further, bulk transcriptomic results can potentially be confounded by transcriptomic signatures arising from other cells, particularly proximal tubular cells, which are the predominant cell type in kidney biopsy samples (2).

Single-cell and single-nucleus RNA sequencing (sc/sn RNA-seq) approaches overcome this limitation by providing transcriptomic data for individual cells, albeit with less depth of coverage than the bulk RNA sequencing (RNA-seq). Application of these single-cell methods to glomerular diseases has enabled investigators to identify cell types within tissues and to characterize the cell injury and activation states and the specific gene expression patterns. Recent studies of scRNA-seq performed on urinary cells and skin lesion biopsies in lupus nephritis reported gene expression profiles that were highly correlated with single-cell data from renal biopsy samples. These findings open up the possibility of alternatives to renal biopsy to establish diagnosis and monitor disease activity (3–5). However, compared to secondary glomerular diseases, relatively few single-cell studies have been published to date in the field of primary glomerular diseases (Table 1).

**Table 1:** Summary of published glomerular disease studies applying single-cell/nucleus technology.

**Disease**	**Author (references)**	**Sample type**	**Sample number**	**Single-cell technology**	**Number of cells & genes/cell analyzed**	**Cell types focused in analysis**	**Main findings**
Lupus nephritis	Arazi et al. (3)	Kidney biopsy and urine	24 lupus nephritis and 10 controls	Single-cell (CEL-seq2 and 10x)	2,736 leukocytes and 145 epithelial cells, 250–5,000 genes per cell	Immune cells	- Total 21 subsets of myeloid, T, NK and B cells with interferon response. - Gene expression of immune cells in urine and kidney was highly correlated.
Lupus nephritis	Der et al. (4, 5)^*^	Kidney biopsy, skin biopsy and urine	21 renal tissue samples and 17 skin biopsy samples from lupus nephritis subjects, three pairs of kidney and skin biopsy samples from controls	Single-cell (Fluidigm C1 mRNA seq HT IFC)	4,019 cells, >100 genes per cell	Tubular cells and skin keratinocytes	- Type I interferon-response signature in tubular cells and skin keratinocytes associated with failure to respond to treatment. - Proliferative subtype is associated with pathways of inflammation and fibrosis which were not observed for membranous subtype.
Diabetic nephropathy	Wilson et al. (6)	Nephrectomy samples	Three diabetic nephropathy and three control samples	Single-nucleus (10x)	23,980 cells, 2,541 genes per cell	Podocytes, mesangial, endothelial, leukocytes, proximal tubules	- Diabetic thick ascending limb, late distal convoluted tubule, and principal cells all adopt a gene expression signature consistent with increased potassium secretion and decreased paracellular calcium and magnesium reabsorption. - Strong angiogenic signatures in glomerular cell types, proximal convoluted tubule, distal convoluted tubule, and principal cells.
Diabetic nephropathy	Abedini et al. (7)	Urine	17 urine samples from five subjects	Single-cell (10x)	23,082 cells, >300 genes per cell	Podocytes, proximal tubules, loop of Henle and collecting duct cells, macrophages, and lymphocytes	- Urine contains the major kidney cell types and leukocytes. - Urinary cells have similar gene expression with kidney cells.
FSGS	Menon et al. (8)	healthy kidney single-cell samples and FSGS glomerular bulk transcriptomic data	24 healthy single-cell samples and 74 FSGS bulk samples	Single-cell (10x)	22,268 cells, 500–5,000 genes per cell	Endothelial cells	- Highest glomerular endothelial cell scores were observed in patients with FSGS. - Glomerular A2M transcript levels associated with lower proteinuria remission rates in FSGS.
FSGS	Latt et al. (9)	Urine from FSGS subjects and bulk transcriptomic data from FSGS and MCD	17 urine samples from 12 subjects captured into 23 single-cell samples	Single-cell (10x)	5,551 cells, 100–6,000 genes per cell	Immune cells and podocytes	- Urine single-cell data showed podocytes showing with inflammatory signatures suggesting EMT, and immune cells, mostly monocytes polarized into M1 and M2 subtypes. - Immune- and EMT-specific genes from FSGS urine single-cells showed higher levels of expression in FSGS compared to MCD samples in NEPTUNE biopsy transcriptomic data.
IgAN	Zheng et al. (10)	Kidney biopsy from IgAN, normal tissue from nephrectomy subjects and peripheral blood mononuclear cells	13 IgAN and six normal kidney samples, five PBMC samples from IgAN and five from healthy donors	Single-cell (STRT-seq)	2,785 kidney cells and 835 PBMCs, more than 3,000 genes/cell	Mesangial, intercalated and principal cells, PBMCs	- IgAN mesangial cells show upregulation of *JCHAIN* which is important for dimerization and transepithelial transportation of IgA. - Peripheral blood monocytes express type I interferon response encoding genes and CD8+ T cells show downregulation of cytotoxic marker genes. - A transitional cell type with intercalated and principal cell markers show EMT and fibrosis signatures.

* *Since the main findings from two studies are similar, we focused on the later study, which has bigger sample size and contains control samples*.

In this review, we will emphasize the recent progress made by single-cell studies of glomerular diseases, particularly lupus nephritis (LN), diabetic nephropathy (DN), focal segmental glomerulosclerosis (FSGS) and IgA nephropathy (IgAN), in understanding pathophysiology at the cellular level and in exploring non-invasive approaches for clinical application. We will also discuss how this approach could be applied to other primary glomerular diseases.

## Single-Cell/Nucleus Studies In Secondary Glomerular Diseases

Secondary glomerular diseases arise as complications of systemic disease, such as systemic vasculitis, systemic lupus, and diabetes. Single-cell/nucleus RNA-seq studies, using kidney biopsy and/or urine samples, have been reported for lupus nephritis and diabetic nephropathy (3–7). ScRNA-seq captures the transcripts from whole cells but it requires enzymatic dissociation of tissue to yield a single-cell suspension, a process that typically causes cellular stress and with that, potentially, transcriptional alterations. Further, this approach may fail to include certain cell types, especially rare cell types, due to incomplete dissociation of kidney tissue (11, 12). Although snRNA-seq captures only nuclear transcripts, it usually covers all the different cell types in the tissue. Both single-cell RNA-seq and single-nucleus RNA-seq approaches yield insights into the tissue composition, cellular mechanisms and provide a detailed, high-resolution assessment of cellular transcriptomics, which goes beyond what can be attained from bulk tissue RNA studies.

## Lupus Nephritis

Studies of bulk transcriptomic analysis in mouse and human LN have reported molecular signatures related to inflammation and fibrosis. Cross-species joint network analysis of human LN and mouse bulk transcriptomic data had identified shared pathways of immune cell infiltration and activation, macrophage and dendritic cell activation, endothelial cell activation, and damage and tissue remodeling/fibrosis in kidney (2, 13).

Details of the immune cell profile of LN were reported in 2019 by Arazi et al., in a single-cell RNA-seq study of immune cells from urine samples and kidney biopsy of LN subjects from the Accelerating Medicines Partnership rheumatoid arthritis/systemic lupus erythematosus (AMP RA/SLE) consortium (3). Stepwise clustering of immune cells identified finer subclusters including inflammatory, phagocytic and M2-like macrophages in the myeloid lineage; central and effector memory T cells, cytotoxic and memory T cells in the T cell lineage; and activated B cells and plasma cells in the B cell lineage. There was a high concordance of gene expression for particular leukocyte classes between urine and biopsy samples, which suggests that most types of immune cells infiltrating the kidneys may also be found in the urine. This concordance supports the potential utility of urine single-cell studies, as they can inform on ongoing inflammatory processes in the kidneys and offers a rationale to pursue similar studies of urine cells in other kidney diseases.

In other reports from the AMP RA/SLE consortium, Der et al. described the upregulation of type I interferon-responsive signature genes in renal tubular cells and in skin keratinocytes. These findings suggest that skin keratinocytes gene signatures could help with assessing the nephritis activity in lupus and serve, in addition to urine cells, as a potential surrogate approach to renal biopsy (4, 5). Moreover, the authors observed differences in the activated pathways between membranous and proliferative subtypes of LN in tubular cells and keratinocytes. In proliferative LN, these cells showed higher expression of genes in pathways of tumor necrosis factor (TNF)-related pathways and interferon response pathways, which were not observed in cells from membranous LN. Consequently, the potential utility of these cellular gene signatures in identifying disease subtypes should be further evaluated. Interferon response signatures and fibrotic signatures were also higher in tubular cells of treatment responder group compared to the non-responder group. These signatures suggest the presence of active innate immune pathways at the time of renal biopsy. This information could be useful to clinicians, as it suggests that these analyses could help verify when effective immunosuppression has been achieved in the kidney.

Interaction analysis showed possible interactions of cytokines across various cell types including cytokines from the TNF (tumor necrosis factor) superfamily, *TNF, TNFSF10* (encoding TNF-related apoptosis-inducing ligand or TRAIL) and *TNFSF14* (encoding LIGHT, homologous to **L**ymphotoxin, exhibits **I**nducible expression and competes with HSV **G**lycoprotein D for binding to **H**erpesvirus entry mediator, a receptor expressed on **T** lymphocytes). These cytokines and related pathways may be potential targets for immunotherapy in LN.

## Diabetic Nephropathy

Bulk transcriptomic studies of DN have identified upregulation of genes that are also recognized in single-cell studies as markers of specific cell types and fibrotic pathways. Lindenmeyer et al. identified elevated tubulointerstitial expression levels of extracellular matrix related genes, including *COL1A2, COL4A1, FN1, VIM1*, and *TIMP1* (14). Similarly, Woroniecka et al. also reported the upregulated expression of collagen genes in renal tubules in a study of human glomeruli and tubules isolated from diabetic subjects. Further, expression of podocyte marker genes, such as *NPHS1, NPHS2, SYNPO, PODXL, WT1*, and *PLA2R1* were downregulated in the glomeruli, which could be due to podocyte dedifferentiation or podocyte loss as a result of inflammation and/or mechanical stress from hyper-filtering glomeruli (15).

Using single-nucleus RNA-seq on cryopreserved kidney biopsy samples, Wilson et al. reported gene expression profiles for various cell types in early human diabetic nephropathy (6). Differential expression tests showed modest values of log-fold changes but identified increased expression of extracellular matrix component collagen genes in mesangial and endothelial cells. Endothelial cells also showed increased expression of glucose transporters and angiogenesis regulators. Notably, interaction analysis identified increased expression of *CCN1*, encoding cellular communication network factor 1, by mesangial cells, as well as possible interactions between integrins and their ligands, which were also upregulated in podocytes and endothelial cells. These predicted interaction pairs appear to suggest on-going cycles of cell injury, tissue repair and fibrosis in DN.

Moreover, gene expression data from these diabetic subjects suggested that tubules may be engaged in greater potassium secretion and decreased calcium and magnesium reabsorption in the thick ascending limb of the loop of Henle and late distal convoluted tubules. There were also infiltrating monocytes and lymphocytes, with monocytes showing an interferon-γ signaling signature.

In a recent report, Abedini et al. captured urinary cells from subjects with DN. They identified a variety of cell types including renal epithelial cells and immune cells, and showed that these cells retained transcriptomic signatures similar to the corresponding cell types in the kidneys (7). The authors found that expression of genes that had previously been nominated to mediate the effect of the polygenic genome-wide association study (GWAS) of estimated glomerular filtration rate (eGFR) were strongly enriched in urinary proximal tubules. These findings connecting the GWAS results with single-cell transcriptomic data may support the notion that proximal tubule activities may be related to eGFR decline in progressive kidney disease, whether as drivers or responders to that decline. As the urine of healthy individuals contains few immune or kidney epithelial cells, a comparison between the gene expression profiles of DN urinary cells and those of urinary cells from healthy individuals was not feasible. However, this study provides proof of the concept that the urine of DN subjects contain cells whose transcriptional profiles may reflect pathological process ongoing in the kidneys.

## Primary Glomerular Diseases

In primary glomerular diseases, intra-renal pathology initiates from within glomerular cells. There are four most common primary glomerular diseases: minimal change disease (MCD), focal segmental glomerulosclerosis (FSGS), membranous nephropathy (MN), and IgA nephropathy (IgAN). Bulk transcriptomic studies have been reported for three of these diseases, (MN is currently an exception), and a few single-cell studies have been reported for FSGS and IgAN. Due to the similar clinical presentations and challenges in making a definitive diagnosis in pediatric patients, FSGS and MCD are often investigated comparatively in transcriptomic studies.

## Focal Segmental Glomerulosclerosis and Minimal Change Disease

FSGS and MCD are primary podocyte diseases that may share similar clinical presentations. In adults, the differential diagnosis is broader, as it includes membranous nephropathy and occasionally other diseases; therefore, establishing a definitive diagnosis usually requires a kidney biopsy. In children, the common diagnostic possibilities are most often limited to MCD and FSGS and so a trial of glucocorticoids is usually undertaken, as the former is uniformly treatment-sensitive and the latter is often treatment-resistant. Moreover, due to the focal nature of lesions in FSGS, early FSGS can be mistaken for MCD if the glomerular lesions are not captured in the biopsy samples. Single-cell and single-nucleus methods could be helpful to elucidate intraglomerular pathophysiology and to identify gene expression signatures of cellular injury, especially in podocytes.

Several bulk tissue transcriptomic studies investigating FSGS and MCD have been reported recently (1, 2, 16–20). The FSGS kidney transcriptomic studies showed the upregulation of inflammatory genes (*SPP1, VCAM1, THY1, CXCL1, CXCL2*, and *CXCL4*) and fibrotic genes (*COL1A1*), none of which were observed in MCD. Using transcriptomic data from the Nephrotic Syndrome Study Network (NEPTUNE) cohort, Sampson et al. reported upregulation of *CXCL9, CXCL11* and *UBD* in the glomerular compartment of subjects with two *APOL1* risk alleles, which are known to have a large effect size in inducing FSGS in African-descent populations, compared to subjects with one or zero risk alleles (1). This suggests that the *APOL1* high risk subjects have a higher degree of inflammation in the glomeruli, although this finding may well have been influenced by the fact that more than half of *APOL1* high risk samples in the analysis had FSGS and the majority of MCD samples, which typically lack glomerular inflammation, had low risk *APOL1* genotypes.

Using data from the NEPTUNE study, the Kidney Precision Medicine Project (KPMP) and the European Renal cDNA Bank (ERCB), Menon et al. derived cell-specific gene expression signatures of healthy human kidney tissue single-cell data and investigated those signatures in bulk kidney cortex transcriptomic data from NEPTUNE for correlation with clinical outcomes. High α-2 macroglobulin (A2M) gene expression from the glomerular endothelial cell signature at the time of initial biopsy was associated with lower FSGS remission rates (8).

In a recent single-cell study of urine samples from FSGS subjects, we reported inflammatory signatures in urine podocytes and immune cells (9). Among the most highly expressed genes in urine podocytes are the genes associated with epithelial-to-mesenchymal transition (EMT). The majority of immune cells were monocytes, polarized into M1 (inflammatory) and M2 (anti-inflammatory) subtypes. EMT is known to predispose to fibrosis, and together with inflammatory signatures from immune cells, could represent a profibrotic signature that could help to differentiate FSGS from MCD. We confirmed this hypothesis using gene expression data from NEPTUNE consortium, which showed higher expression of immune and EMT genes in FSGS compared to MCD samples. Immune genes were also found to be higher in NEPTUNE nephrotic syndrome cases without remission compared to cases with remission.

Cell-cell interaction analysis revealed possible interaction of *TNFSF12* (encoding tumor necrosis factor-like weak inducer of apoptosis or TWEAK) with *TNFRSF12A* (Fn14) and *TNFSF10* (TRAIL) with *TNFRSF10B* (DR5) and with *TNFRSF11B* (osteoprotegerin) receptors between the immune cells and kidney epithelial cells. Both cytokines are known to be involved in apoptosis, chronic inflammation, organ remodeling and fibrosis (21–28), and their signaling pathways could be targets for immunotherapy. This study suggests that urine testing could provide valuable information for differential diagnosis of nephrotic syndrome in the pediatric population as a form of liquid biopsy and might contribute to disease activity monitoring in children and adults.

## IgA Nephropathy

IgAN is caused by overproduction of immunoglobulin A1 (IgA1) and deposition of IgA1-IgG immune complexes in glomeruli with subsequent mesangial proliferation and extracellular matrix deposition (29). Hodgin et al. compared the bulk transcriptomes of IgAN glomeruli with and without endocapillary proliferation. There was upregulation of genes related to classical complement activation (*C1QA, C1QB, C2*, and *VSIG4*), extracellular matrix degradation and turnover (*HSPE, TIMP1*) and *CD163*, which is a marker of M2 macrophage polarization, in glomeruli with endocapillary proliferation and these genes were inversely correlated with renal function (30).

Zheng et al. recently reported the first single-cell gene expression profile of kidney cells and peripheral blood monocytes from IgAN (10). The authors isolated glomerular cells, tubular epithelial cells and immune cells in a stepwise manner and captured mesangial cells and a smaller number of podocytes for single-cell analysis. The IgAN mesangial cells showed upregulation of *JCHAIN* which is essential for dimerization and transepithelial transportation of IgA, and the peripheral blood monocytes showed significant expression of type 1 interferon-encoding genes. Cell-cell interaction analysis showed increased interactions in IgAN mesangial and endothelial cells and reduced interactions in intercalated cells compared to their healthy counterparts. Further analysis of intercalated cells revealed a new transitional cell type which expressed marker genes of both intercalated and principal cells and genes associated with EMT.

## Discussion

For an increasing number of kidney diseases, single-cell technology has provided insights into the cellular landscape of inflammation, the molecular crosstalk among various immune, epithelial and mesenchymal cell types, and the gene expression profiles of these cells. Cellular injury patterns may well-reflect the effect of immune or inflammatory response superimposed on the primary disease mechanisms. Single-cell analysis of four different mouse kidney disease models (nephrotoxic serum nephritis, diabetes, doxorubicin toxicity, and CD2AP deficiency) showed cell type-specific and injury type-specific response in glomeruli (31). It remains to be seen whether and to what extent single-cell approaches can help to establish the primary etiology in particular cases of human primary glomerular diseases since there are no published single-cell/nucleus study on kidney biopsy samples from FSGS, MCD, or MN to date.

As discussed above, urine single-cell data from lupus nephritis, diabetic nephropathy and FSGS studies demonstrate that urine contains immune and kidney epithelial cells that reflect the intrarenal pathology. For the purpose of distinguishing FSGS from MCD in the diagnosis of pediatric nephrotic syndrome, studies with larger sample sizes will be needed to capture urine immune cells, possibly by fluorescence-activated cell sorting (FACS) and/or to quantify the protein products of key inflammatory genes from immune cells in serum and urine by enzyme-linked immunosorbent assay (ELISA) and determine the sensitivity and specificity of these approaches.

For some primary glomerular diseases, podocytes are the initial loci for key pathologic processes. The digestion of glomeruli from tissue in a single-cell approach is usually not complete and some kidney single-cell studies did not capture the podocytes (4, 32). In FSGS and membranous nephropathy biopsies, particularly when advanced, it will be more challenging to release cells from sclerosed or partially sclerosed glomeruli. In these cases, the single-nucleus RNA-seq approach is more effective than single-cell RNA-seq in enriching for podocytes (33). Technical challenges in designing single-cell/single-nucleus experiments with currently available protocols was discussed in detail in an informative recent review by Deleersnijder et al. (34).

For FSGS, characterizing differences in cell type composition and the transcriptional profiles among cells of a single type and comparing sclerosed and normal-appearing glomeruli could be important in understanding the focal and the segmental nature of the lesions. One strategy to address this could be spatial transcriptomics, which localizes RNA expression patterns within tissue. This technique has not yet been applied widely in the field of nephrology, but it may provide insight into the focal and segmental nature of FSGS, the origin of myofibroblasts and the molecular crosstalk among various cell types in the glomerular microenvironment even at the current resolution limits.

An important pathophysiological process that must be addressed in glomerular diseases is progressive renal fibrosis. IgAN and FSGS urine single-cell studies report expression of genes associated with EMT in epithelial cells, such as podocytes in FSGS and principal and intercalated cells in IgAN. However, a recent single-cell study of subjects with hypertensive nephrosclerosis with and without chronic kidney disease (CKD) found that the contribution of epithelial cells (de-differentiated proximal tubule cells) to fibrosis by EMT could be relatively minor compared to mesenchymal cells (35). Notably, the authors enriched non-proximal tubule cells to study them separately from proximal tubule cells, which are the major cell type in renal cortex samples. Myofibroblasts are defined in the study as the cell type that expressed the most extracellular matrix (ECM). Diffusion mapping analysis, which organizes single-cell data along complex pathways that may provide insight into cell lineages, suggests fibroblasts and pericytes as the major cellular source of myofibroblasts in hypertensive CKD. The extent of contribution of EMT to sclerosis and the origin of myofibroblasts in other glomerular diseases remain to be identified.

Another avenue to be explored for understanding primary glomerular diseases is to identify the immunological triggers that lead to renal diseases. Genetic studies indicate strong associations with immune loci, especially the class II major histocompatibility complex (MHC) genes, for IgAN, idiopathic MN and childhood steroid sensitive nephrotic syndrome (36–42). Single-cell studies of peripheral blood mononuclear cells (PBMCs) may illuminate immune signatures associated with disease onset or relapse, and in the case of minimal change disease, may allow to identify putative plasma factors secreted by dysregulated or activated immune cells leading to podocyte injury. A recent study of PBMCs from subjects who received immunization used cellular indexing of transcriptomes and epitopes by sequencing (CITE-seq) to profile 82 surface proteins of these immune cells (43). The authors reported that differences in baseline blood transcriptional signatures are predictive of antibody response to influenza and yellow fever vaccination, and that these signatures reflect the extent of activation in plasmacytoid dendritic cells and lymphocytes. These same signatures were also associated with disease activity in patients with lupus with plasmablast-associated flares. CITE-seq is a powerful approach to identify novel immune cell activation states and to characterize cell subtypes with confidence; it offers opportunities to study the immunological basis of glomerular diseases.

Single-cell technology can also be used to identify culprit genes and cell types from among the wealth of candidates identified by GWAS studies. Many GWAS-identified disease loci are located in non-coding intronic or intergenic genomic regions and yet are associated with gene expression levels in tissues (44, 45). In this case, it can be challenging to identify the critical causal genes and cell types in order to fully interpret the results.

Single-nucleus assay for transposable chromatin with sequencing (ATAC-seq) can give information about regions of open chromatin state in a particular cell at a particular moment. Further, it can identify cell types that harbor enhancer elements in the regions encompassing the most highly disease-correlated single nucleotide polymorphisms (SNPs). Together with sc/sn RNA-seq data in high risk and low risk genotype groups, ATAC-seq might help to pinpoint the causal genes for those GWAS hits that confer disease risk by regulating gene expression.

Ongoing innovations in the single-cell technology and analysis offer better prospects to identify important cell types and states in kidney diseases. Using pseudotime analysis of single-nucleus RNA-seq and ATAC-seq data from developing and adult mouse kidneys, Miao et al. delineated the trajectory of the developmental process in mice from nephron progenitor cells to podocytes and renal tubular cells (46). Pseudotime analysis may be applied to human disease data to characterize the differentiation and transition processes from one cell state to another in the presence or absence of treatment or in longitudinal studies. Cell-cell interaction analysis can also provide insight into intercellular signaling in the glomerular microenvironment and identify potential biomarkers and therapeutic targets. In conclusion, the single-cell technology represents a powerful tool to dissect pathophysiology and can be exploited to provide new insights into glomerular disease pathogenesis.

## Author Contributions

KL reviewed the literature and drafted the manuscript. JK, JH, and TY provided comments. KL and JK edited the manuscript. All authors read and approved the final draft.

## Funding

This work was supported by the NIDDK Intramural Research Program, under project ZO1 DK-043308.

## Conflict of Interest

The authors declare that the research was conducted in the absence of any commercial or financial relationships that could be construed as a potential conflict of interest.

## Publisher's Note

All claims expressed in this article are solely those of the authors and do not necessarily represent those of their affiliated organizations, or those of the publisher, the editors and the reviewers. Any product that may be evaluated in this article, or claim that may be made by its manufacturer, is not guaranteed or endorsed by the publisher.
